# Integrated microbiome and metabolomics analysis reveals the alleviating effect of *Pediococcus acidilactici* on colitis

**DOI:** 10.3389/fvets.2025.1520678

**Published:** 2025-02-26

**Authors:** Lulu Wu, Lixun Xue, Xin Ding, Huyan Jiang, Ranran Zhang, Aifang Zheng, Yuan Zu, Shuaishuai Tan, Xin Wang, Zhigang Liu

**Affiliations:** ^1^School of Life Sciences, Anqing Normal University, Anqing, China; ^2^School of Chemistry and Chemical Engineering, Anqing Normal University, Anqing, China; ^3^Engineering Technology Research Center for Aquatic Organism Conservation and Water Ecosystem Restoration in University of Anhui Province, Anqing, China; ^4^Key Laboratory of Biodiversity Conservation and Characteristic Resource Utilization in Southwest Anhui, Anqing, China; ^5^Anqing Forestry Technology Innovation Research Institute, Anqing, China

**Keywords:** *Pediococcus acidilactici*, colitis, gut microbiota, metabolite, arachidonic acid metabolism

## Abstract

Colitis is a complicated disease caused by multiple factors, seriously threatening the host health and the development of animal husbandry. Probiotics have been demonstrate to participate in the active regulation of multiple gastrointestinal disease, gut microbiota and metabolism, but research on the efficacy of *Pediococcus acidilactici* isolated from dogs in alleviating colitis remains scarce. Here, we aimed to investigate the ameliorative effects of *Pediococcus acidilactici* isolated from dogs on colitis induced by LPS and its underlying molecular mechanisms. For this purpose, we collected colon contents from 15 mice for amplicon sequencing and metabolic analysis. Results showed that *Pediococcus acidilactici* could relieve the colon damage and cytokine disorder caused by colitis. Microbiome analysis showed that colitis could cause a significant decrease in the gut microbial diversity and abundance, but *Pediococcus acidilactici* administration could restore the microbial index to the control level. Metabolomics analysis showed that 8 metabolic pathways and 5 (spermine, L-Arginine, 15-Deoxy-Delta12,14-PGJ2, prostaglandin J2, and 15(S)-HETE) metabolites may be involved in the alleviation of colitis by *Pediococcus acidilactici*. In summary, these findings demonstrated that the positive regulation effect of *Pediococcus acidilactici* on gut microbiota and metabolism may be one of its underlying mechanisms to alleviate colitis. Additionally, this study also conveyed a vital message that *Pediococcus acidilactici* isolated from dogs may serve as a promising candidate to ameliorate *Pediococcus acidilactici*.

## Introduction

The intestine harbors approximately 10^14^ microbial cells involving over 2,000 distinct species (1–3). These gut-inhabiting microbes, also known as gut microbiota, have been showed to function in host health, metabolism, intestinal homeostasis and intestinal barrier maintenance (4–9). Additionally, the gut microbiota is also essential participant and maintainer of the intestinal mucosal barrier, which play key roles in preventing pathogen invasion and maintaining intestinal homeostasis (6, 7, 10). Gut microbial community, as crucial biochemical converters, can transform the complex chemical space presented by nutrition and diet into the metabolite environment (11, 12). These metabolites including cholic acid, indole derivatives and short-chain fatty acids (SCFAs) participate in the positive regulation of the host health and intestinal homeostasis by acting on the intestine or other organ systems (13). However, gut microbial homeostats is susceptible to external factors, especially gastrointestinal related diseases (1, 14).

Inflammatory bowel disease (IBD) is a chronic intermittent disease primarily affecting the rectal and colon mucosa (15, 16). It is characterized by intestinal inflammation and damage to the epithelial barrier (17). IBD has gained significant attention in recent years due to its detrimental impact on host health. The prevalence of IBD exceeds 0.3% in developed countries, and its incidence rate is also gradually increasing in newly industrialized countries. Animals, such as dogs, cats, horses, and dairy cows, are also affected by IBD, leading to substantial economic losses and threats to animal welfare (18, 19). IBD is a complex disease influenced by factors such as diet, stress, genetics, and the environment (20). Recent studies have also linked colitis to gut microbial dysbiosis (21–23). Clinically, antibiotics, steroids, and immunosuppressants are commonly used to treat IBD. However, these therapies have drawbacks, including drug dependence, high cost, and antibiotic resistance, particularly for patients requiring long-term medication (24, 25). Therefore, the discovery of healthy and effective management options for IBD is crucial. Emerging research indicates that the regulation of gut microbiota and its metabolites holds great potential in IBD treatment (26–28). Dietary intervention, particularly the probiotics administration, is currently considered one of the most effective methods for regulating gut microbiota and metabolism (29).

Probiotics are microorganisms, such as *Pediococcus acidilactici*, Bifidobacterium, and *Bacillus subtilis*, that provide benefits to the host when consumed in sufficient amounts (30, 31). Previous studies have demonstrated the positive impact of *Pediococcus acidilactici* on host growth performance, digestive enzyme activity, intestinal villus height, and antioxidant capacity (32–34). Furthermore, *Pediococcus acidilactici* has been found to aintain gut microbial homeostasis and improve intestinal barrier function, suggesting their potential in alleviating gastrointestinal diseases (26, 27, 35). The interaction between probiotics, gut microbiota, and the host has become a significant focus in gastrointestinal disease research. Although there is substantial evidence supporting the alleviative effects of probiotics on colitis, there is a lack of studies specifically investigating the dogs source of *Pediococcus acidilactici*. Therefore, our objective is to evaluate whether *Pediococcus acidilactici* derived from dogs can alleviate colitis by modulating gut microbiota and metabolism. Meanwhile, this research will contribute to the expansion of canine probiotics applications and establish a foundation for the prevention and treatment of colitis using probiotics.

## Materials and methods

### Animals treatment and sample acquisition

In this study, 24 specific pathogen-free (SPF) male Kunming mice (8-week-old, 42–44 g) were randomly divided into three groups following 3 days of adaptive feeding: control group (CON), *Pediococcus acidilactici* treatment group (RSPQ), and the colitis group (DSS). There were 8 mice in each group. The mice were maintained under standard temperature and humidity conditions and provided with a sufficient diet and drinking water. Additionally, from day 1 to day 7, the DSS and RSPQ groups received drinking water supplemented with 3% (w/v) dextran sulfate sodium salt (DSS) to induce colitis. The specific steps of *Pediococcus acidilactici* preparation refer to previous research (36). During days 8–14 of the experiment, the RSPQ group was supplemented with *Pediococcus acidilactici* (0.2 mL, 5 × 10^9^ CFU/mL) that had been prepared in advance, while the DSS and CON groups received an equivalent volume of normal saline. At the conclusion of the experiment on day 15, all mice were euthanized, and colon tissue, colon contents, and serum samples were collected for subsequent analysis.

### 16S rDNA amplicon sequencing

According to previous studies, the DNA of each samples was extracted using a commercial kit (37). We designed universal primers (338F: ACTCCTACGGGAG GCAGCAG-3 and 806R: GGACTACHVGGGTWTCTAAT) and added sequencing adapters for PCR amplification. Subsequently, the amplified products were purified, quantified, and normalized to form sequencing libraries. The DNA was quantified via utilizing UV–Vis spectrophotometer (NanoDrop 2000, United States) and DNA integrity was assessed by 0.8% agarose gel electrophoresis. Sequencing libraries were constructed using PacBio platform (Biomarker Technologies, China) according to the manufacturer’s specifications. The constructed libraries needed to be quality checked (concentration more than 2 nM), and the qualified libraries were sequenced using Illumina Novaseq 6000. The raw image data files were converted into raw sequencing sequences through base calling analysis. Meanwhile, the results were stored in the FASTQ (abbreviated as fq) file format, which contains the sequence information of the sequencing sequences (Reads) and their corresponding sequencing quality information. Moreover, the analysis of gut microbiota included the following operations: (1) Quality control of the original sequencing sequences to remove unqualified data; (2) OTUs clustering and classification based on sequence composition; (3) According to the OTUs results, taxonomic analysis of samples at various taxonomic levels was performed to explore gut microbial composition; (4) Alpha diversity indices were calculated and explore the species diversity within individual sample; (5) Beta diversity analysis was used for comparing gut microbial construction; (6) Statistical analyses were performed using GraphPad Prism (version 9.0c) and R (v3.0.3) software. Differential taxa associated with colitis were identified using Metastats analysis. Data are expressed as mean ± SEM, and statistically significant differences are denoted as *p* < 0.05.

### Histological observations and cytokine analysis

In this study, we prepared tissue sections and HE staining according to previous studies (38, 39). Meanwhile, the IL-6, TNF-α, and IL-1β levels were conducted in accordance with the recommendations of the ELISA kits.

### Metabolomics analysis

To further investigate the effects of *Pediococcus acidilactici* on the intestinal metabolism, we explored changes in intestinal metabolism using untargeted metabolomics. The metabolomic procedure such as sample preparation, metabolite identification, data processing and metabolic pathway analysis were determined as per previous research (9, 40).

## Results

### Histopathological and cytokine analysis

**Figure 1:**
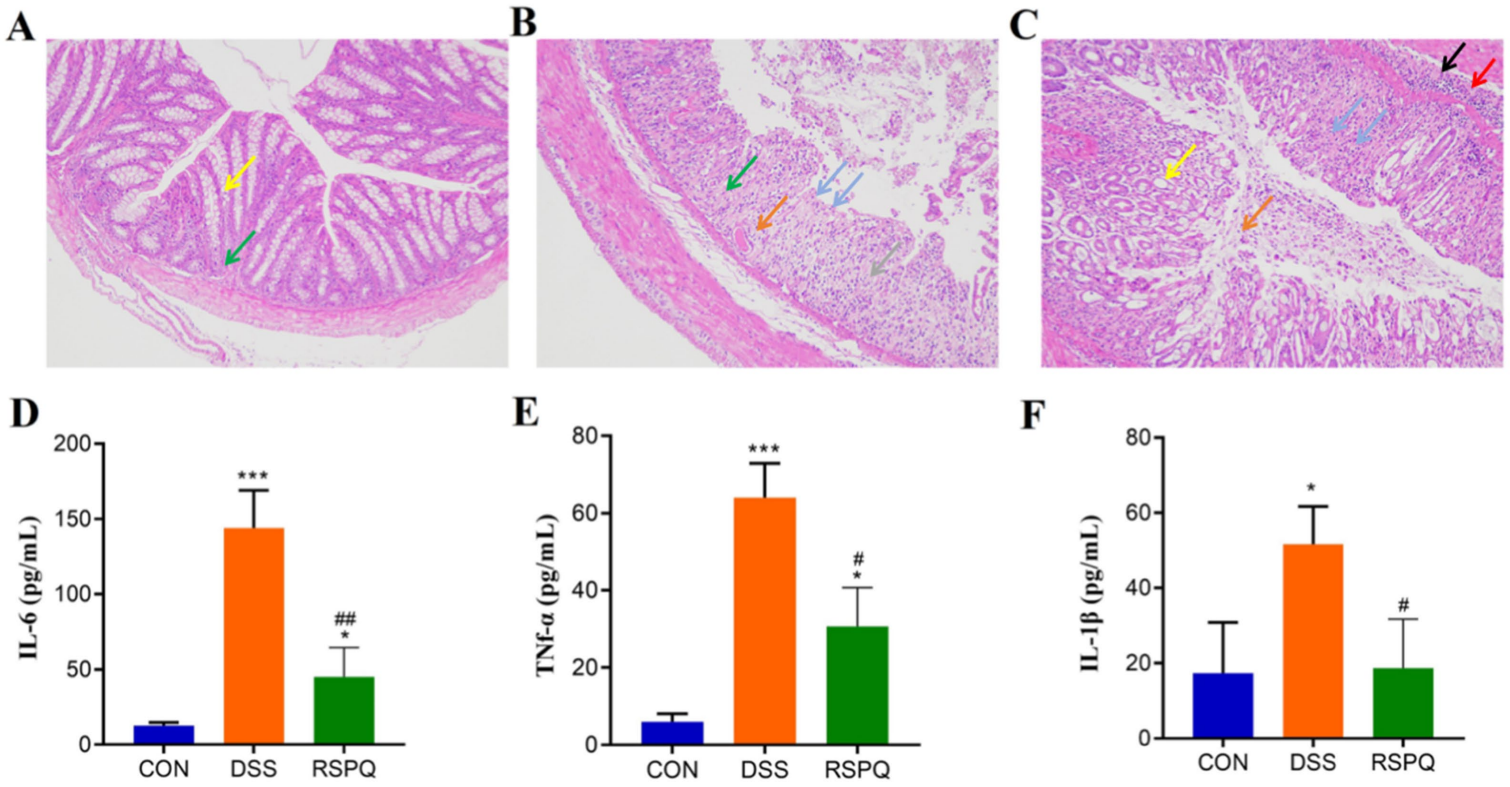
*Pediococcus acidilactici* alleviate intestinal damage and cytokine disorder caused by colitis. **(A–C)** Histopathological observation of colitis in the CON, DSS and RSPQ. **(D–F)** Serum concentrations of IL-6, TNF-α, and IL-1β. *p < 0.05 and ***p < 0.001 vs. the CON, ^#^*p* < 0.05 and ^##^*p* < 0.01 vs. the DSS.

In this research, we observed that *Pediococcus acidilactici* administration can restore colitis-induced weight loss in mice (Supplementary Figure S1). Moreover, the histopathological results of each group are presented in Figures 1A–C. Results indicated that the colon in the CON group was clear and no damage was observed. However, the colon of the DSS group exhibited extensive ulceration, a loss of mucosal and intestinal gland architecture, a reduced number of goblet cells, significant hyperplasia and repair of connective tissue (green arrows), and an abundance of newly formed blood vessels (gray arrows). Furthermore, a notable infiltration of lymphocytes was observed in the lamina propria of the colon tissue in the DSS group (blue arrows), alongside a limited presence of eosinophils in the intestinal glands (orange arrows). However, *Pediococcus acidilactici* administration can reduce the range of ulcers and restore colon damage. Serum cytokine analysis revealed that the levels of IL-6, TNF-α, and IL-1β were significantly increased in the DSS group compared with the CON group (Figures 1D–F). However, *Pediococcus acidilactici* administration could significantly reduce the increase in the levels of the above cytokines caused by colitis.

### *Pediococcus acidilactici* restores alterations in gut microbial diversity associated with colitis

**Figure 2:**
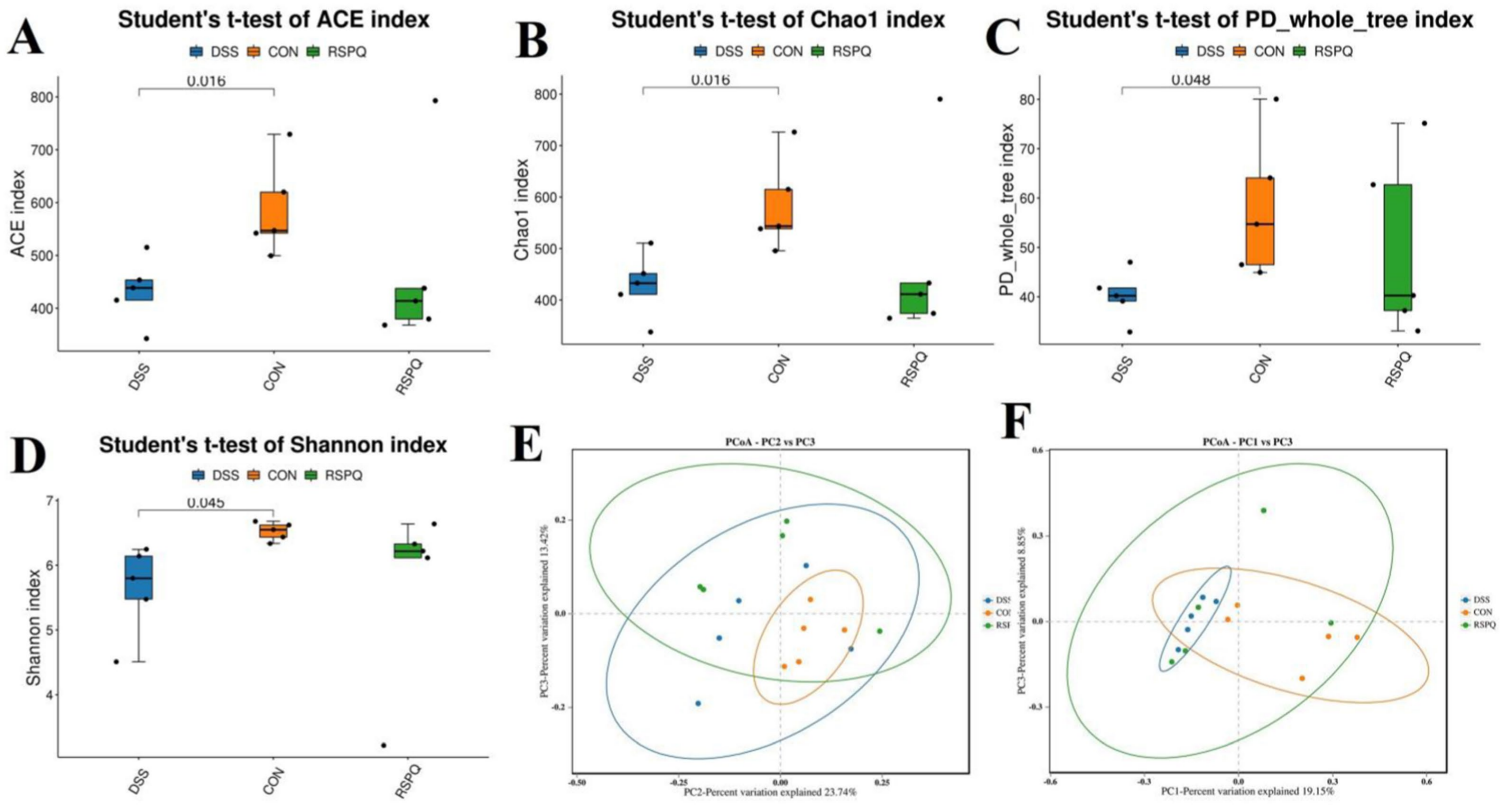
*Pediococcus acidilactici* administration reversed shifts in gut microbial diversity and structure associated with colitis in mice. **(A)** Chao1 index; **(B)** ACE index; **(C)** PD_whole_tree index; **(D)** Shannon index. PCoA plots based on the weighted **(E)** and unweighted **(F)** uniFrac distance. Each point on the graph represents a sample, with distinct colors denoting different groups. The distance between points illustrates the degree of variation among the samples.

In this research, we explored the differences of the gut microbial abundance and diversity by comparing ACE, Chao1, PD_whole_tree and Shannon. There were statistically significant differences in the gut microbial ACE (587.55 ± 40.35 versus 433.04 ± 27.88, *p* < 0.05), Chao1 (583.76 ± 40.46 versus 428.63 ± 28.09, *p* < 0.05), PD_whole_tree (58.05 ± 6.46 versus 40.19 ± 2.27, *p* < 0.05) and Shannon (6.52 ± 0.06 versus 5.63 ± 0.31, *p* < 0.05) indices between DSS and CON groups, whereas the above-mentioned indices were not significantly different between the CON and RSPQ groups (Figures 2A–D). Intergroup analysis intuitively revealed that colitis could significantly decrease gut microbial abundance and diversity, thereby causing gut microbial dysbiosis. However, *Pediococcus acidilactici* administration could restore the gut microbial diversity and abundance to the control level and maintain gut microbial homeostasis. Moreover, PCoA plots generated from the weighted and unweighted UniFrac distances were applied to assess the beta diversity. Results revealed that the individuals in these groups were clustered together, suggesting no significant differences in the gut microbial construction (Figures 2E,F).

### *Pediococcus acidilactici* administration restored gut microbial dysbiosis during colitis

**Figure 3:**
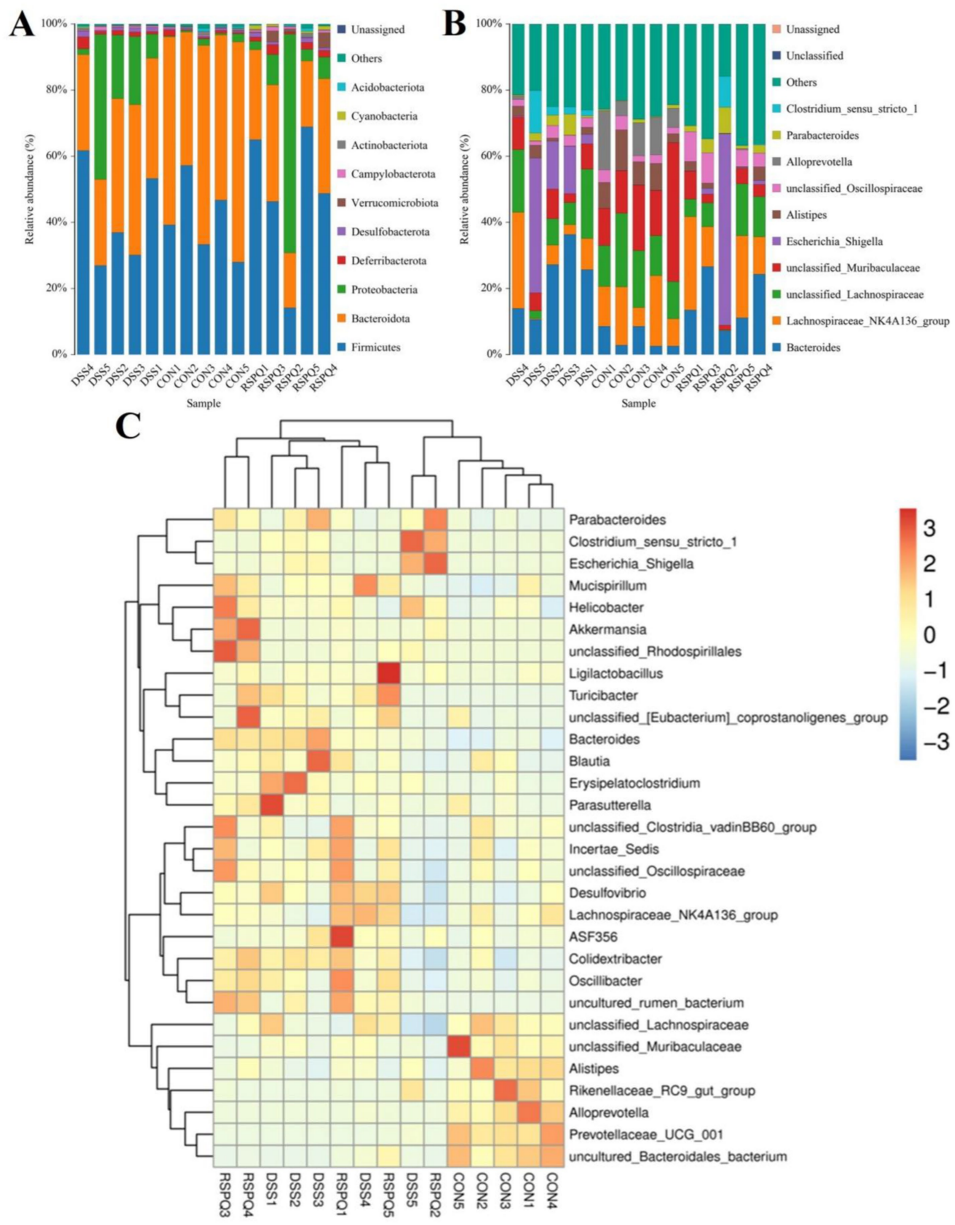
Relative abundance distribution of different samples at the phylum **(A)** and genus **(B)** levels. Only the top 10 most abundant bacterial phyla and genera are displayed in each sample. **(C)** Cluster heatmap of different samples at the genus level. The gradient of blue to red represents the alteration of abundance from low to high.

There were 30 phyla and 571 genera identified from acquired samples, varying from 18 to 26 phyla and 131–299 genera per sample. Specifically, the gut microbiota in the CON, DSS and RSPQ groups were predominated by Firmicutes (CON = 40.93%, DSS = 41.71%, RSPQ = 48.23%), Bacteroidota (CON = 54.74%, DSS = 35.32%, RSPQ = 26.50%) and Proteobacteria (CON = 1.12%, DSS = 18.79%, RSPQ = 18.25%) (Figure 3A). Moreover, other phyla such as Campylobacterota (CON = 0.24%, DSS = 0.50%, RSPQ = 0.64%), Verrucomicrobiota (CON = 0.036%, DSS = 0.024%, RSPQ = 1.79%), Actinobacteriota (CON = 0.48%, DSS = 0.35%, RSPQ = 0.42%), Acidobacteriota (CON = 0.37%, DSS = 0.16%, RSPQ = 0.30%) and Cyanobacteria (CON = 0.053%, DSS = 0.10%, RSPQ = 0.68%) in the CON, DSS and RSPQ groups were represented with a lower abundance. Among recognized genera, unclassified_*Muribaculaceae* (19.87%), unclassified_*Lachnospiraceae* (15.04%) and *Lachnospiraceae*_NK4A136_group (13.00%) were the most prevalent bacteria in the CON group, accounting for approximately 47.92 of overall composition (Figure 3B). Additionally, the dominant bacterial genera observed in the DSS group were *Bacteroides* (22.55%), *Escherichia_Shigella* (14.79%) and unclassified_*Lachnospiraceae* (11.38%), whereas *Bacteroides* (16.31%) was the most predominant bacterial genus in the RSPQ groups, followed by *Lachnospiraceae*_NK4A136_group (15.13%) and *Escherichia_Shigella* (12.85%). Furthermore, clustering heatmap also showed the composition and abundance distribution of gut microbiota in the CON, DSS and RSPQ groups and demonstrated the significant effects of colitis on gut microbiota (Figure 3C).

**Figure 4:**
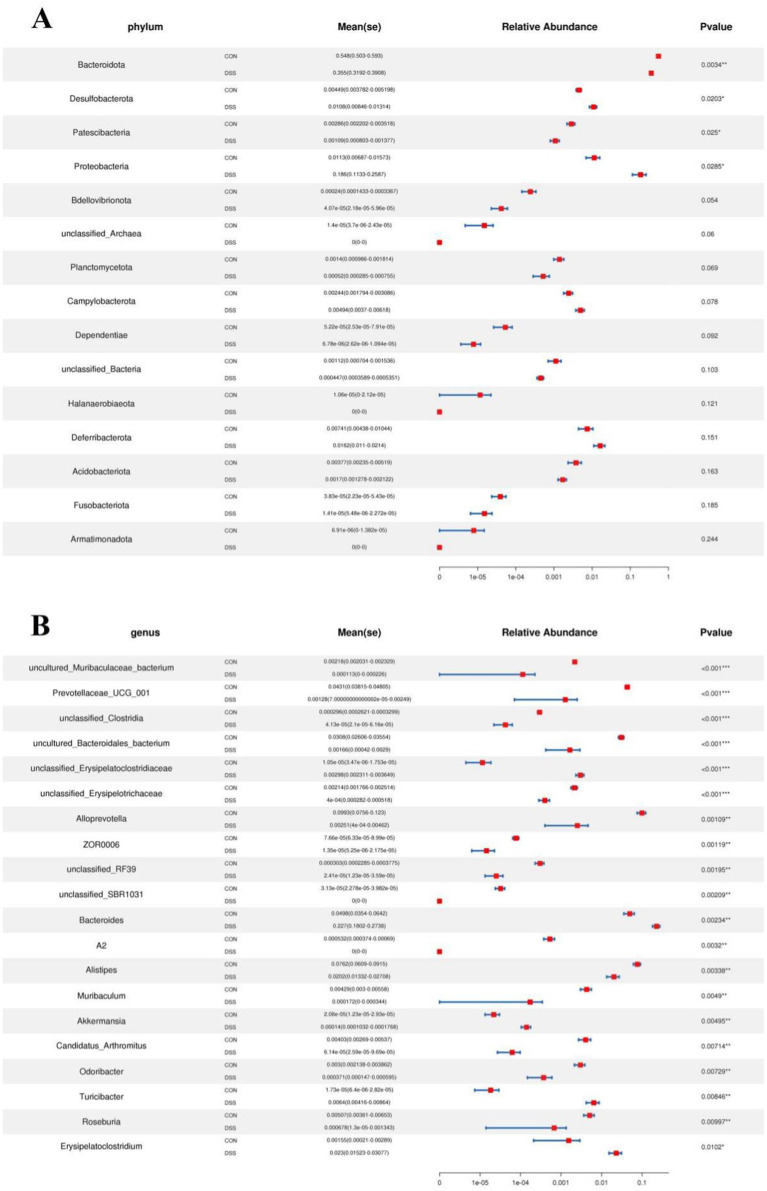
Taxa that were significantly different between the CON and DSS groups at the phylum **(A)** and genus **(B)** levels. All of the data represent means ± SD. *p < 0.05, **p < 0.01, ***p < 0.001.

**Figure 5:**
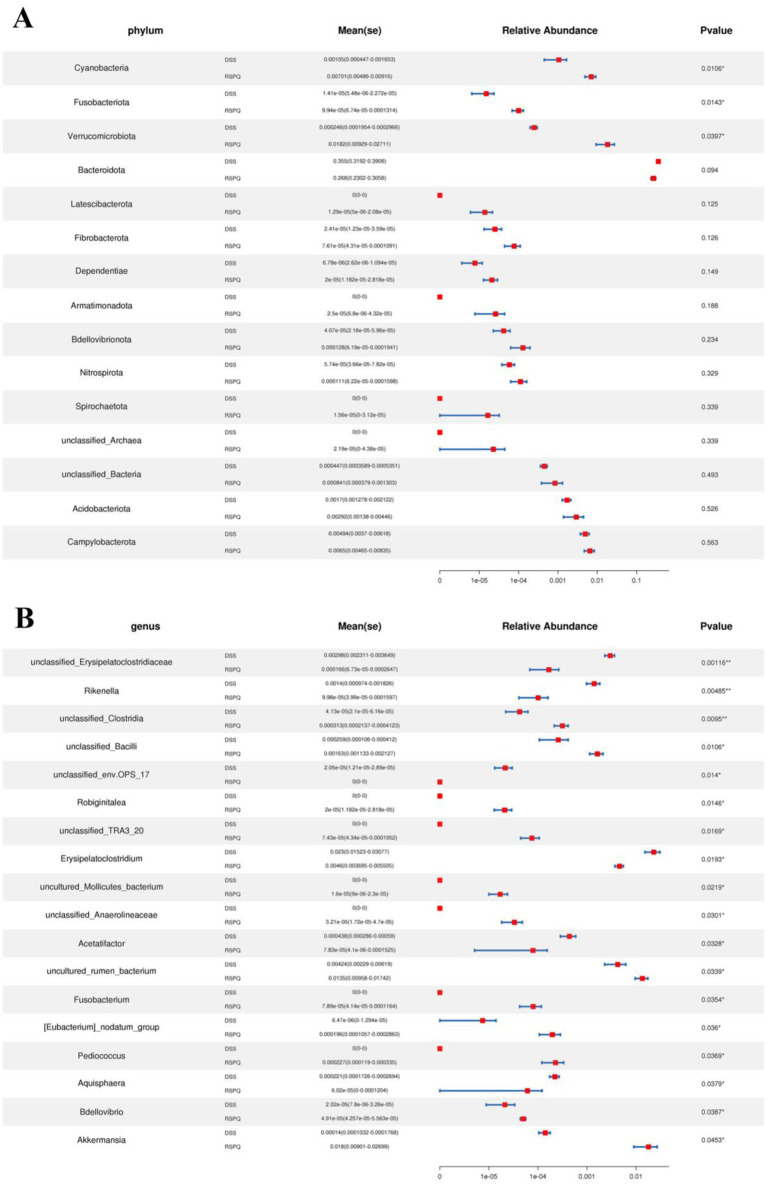
Taxa that were significantly different between the RSPQ and DSS groups at the phylum **(A)** and genus **(B)** levels. All of the data represent means ± SD. *p < 0.05, **p < 0.01.

Metastats analysis was used for distinguishing the differential taxa at different classification levels to further explore the effects of *Pediococcus acidilactici* administration on gut microbiota in mice with colitis. At the phylum level, the gut microbiota in the DSS group exhibited significant increase in the relative proportions of Desulfobacterota and Proteobacteria, whereas Bacteroidota, Patescibacteria and Bdellovibrionota decreased dramatically as compared to CON group (Figure 4A). Moreover, 49 bacterial genera were found to be significantly different between CON and DSS groups. Among them, the relative abundances of 15 bacterial genera (unclassified*_Erysipelatoclostridiaceae*, *Bacteroides*, *Akkermansia*, *Turicibacter*, *Erysipelatoclostridium*, unclassified*_env.OPS_17*, *Streptococcus*, unclassified*_Desulfovibrionaceae*, *Bilophila*, *Romboutsia*, *Enterorhabdus*, *Escherichia_Shigella*, *Enterococcus*, uncultured*_Clostridiales_bacterium*, and uncultured*_rumen_bacterium*) significantly increased, whereas the relative richness of 34 bacterial genera (uncultured*_Muribaculaceae_bacterium*, *Prevotellaceae_UCG_001*, unclassified*_Clostridia*, uncultured*_Bacteroidales_bacterium*, unclassified*_Erysipelotrichaceae*, *Alloprevotella*, *ZOR0006*, unclassified*_RF39*, unclassified*_SBR1031*, *A2*, *Alistipes*, *Muribaculum*, *Candidatus_Arthromitus*, *Odoribacter*, *Roseburia*, *Marvinbryantia*, *[Eubacterium]_nodatum_group*, *Prevotellaceae_NK3B31_group*, unclassified*_Rokubacteriales*, unclassified*_Gaiellales*, *Paenibacillus*, unclassified*_Xanthobacteraceae*, *Sphingomonas*, *Mesoplasma*, unclassified*_Muribaculaceae*, uncultured*_soil_bacterium*, unclassified*_Comamonadaceae*, *Candidatus_Saccharimonas*, unclassified*_BIrii41*, unclassified*_Acetobacteraceae*, unclassified*_Isosphaeraceae*, *Rikenellaceae_RC9_gut_group*, *Anaerotruncus*, and unclassified*_TRA3_20*) memorably decreased during colitis (Figure 4B). At the phylum level, the abundances of Patescibacteria and Fusobacteriota was observably more preponderant in RSPQ than in the DSS, whereas the abundances of Proteobacteria was lower (Figure 5A). Moreover, we observed that the relative abundances of six genera (unclassified*_Erysipelatoclostridiaceae*, *Rikenella*, unclassified*_env.OPS_17*, *Erysipelatoclostridium*, *Acetatifactor* and *Aquisphaera*) obviously decreased significantly increased, whereas the relative abundances of 12 genera (*Iunclassified_Clostridia*, unclassified*_Bacilli*, *Robiginitalea*, unclassified*_TRA3_20*, *uncultured_Mollicutes_bacterium*, unclassified*_Anaerolineaceae*, uncultured*_rumen_bacterium*, *Fusobacterium*, *[Eubacterium]_nodatum_group*, *Pediococcus*, *Bdellovibrio* and *Akkermansia*) significantly increased in RSPQ as compared to DSS (Figure 5B).

### *Pediococcus acidilactici* administration ameliorated intestinal metabolism during colitis

**Figure 6:**
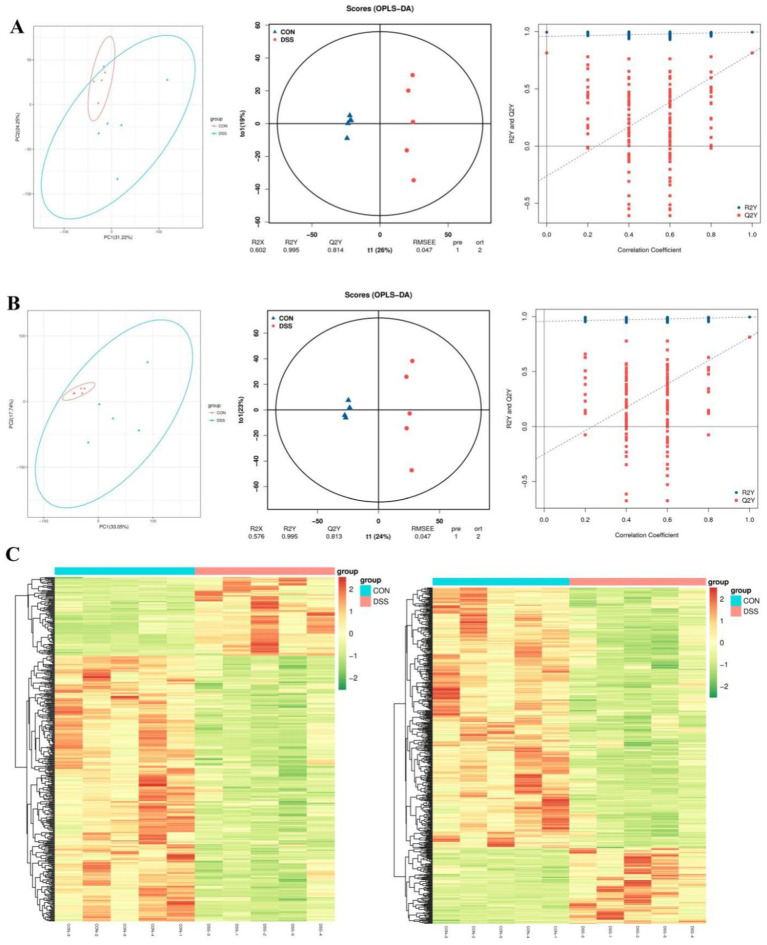
Multivariate statistical analysis of intestinal metabolism in CON and DSS groups. **(A,B)** PCA score plots, OPLS-DA score plots and permutation tests of the OPLS-DA in positive-ion and negative-ion modes. **(C)** Cluster heat map of differential metabolites in CON and DSS groups in positive-ion and negative-ion modes.

**Figure 7:**
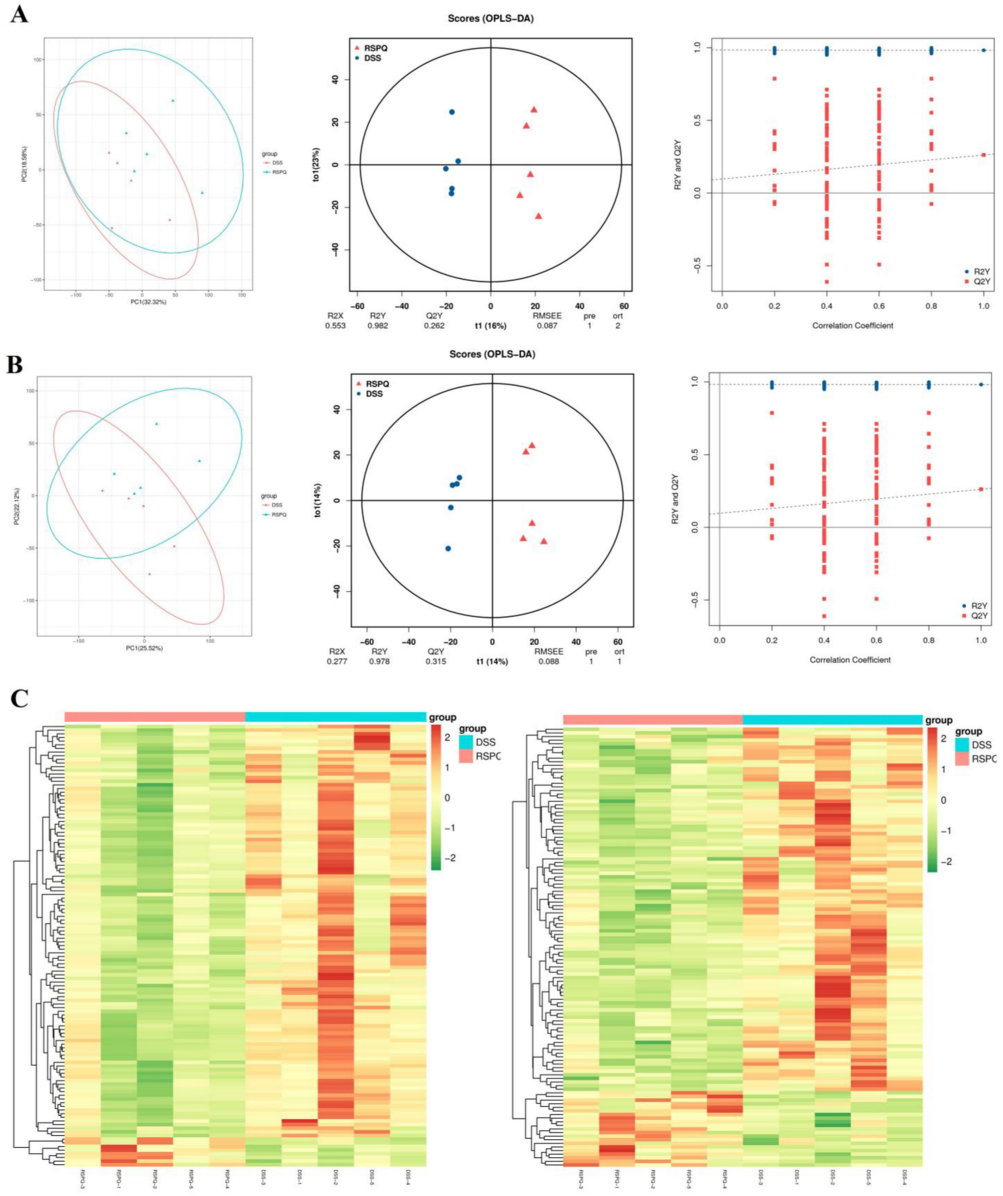
Multivariate statistical analysis of intestinal metabolism associated with *Pediococcus acidilactici* and colitis. **(A,B)** PCA score plots, OPLS-DA score plots and permutation tests of the OPLS-DA in positive-ion and negative-ion modes. **(C)** Cluster heat map of differential metabolites in CON and DSS groups in positive-ion and negative-ion modes.

**Figure 8:**
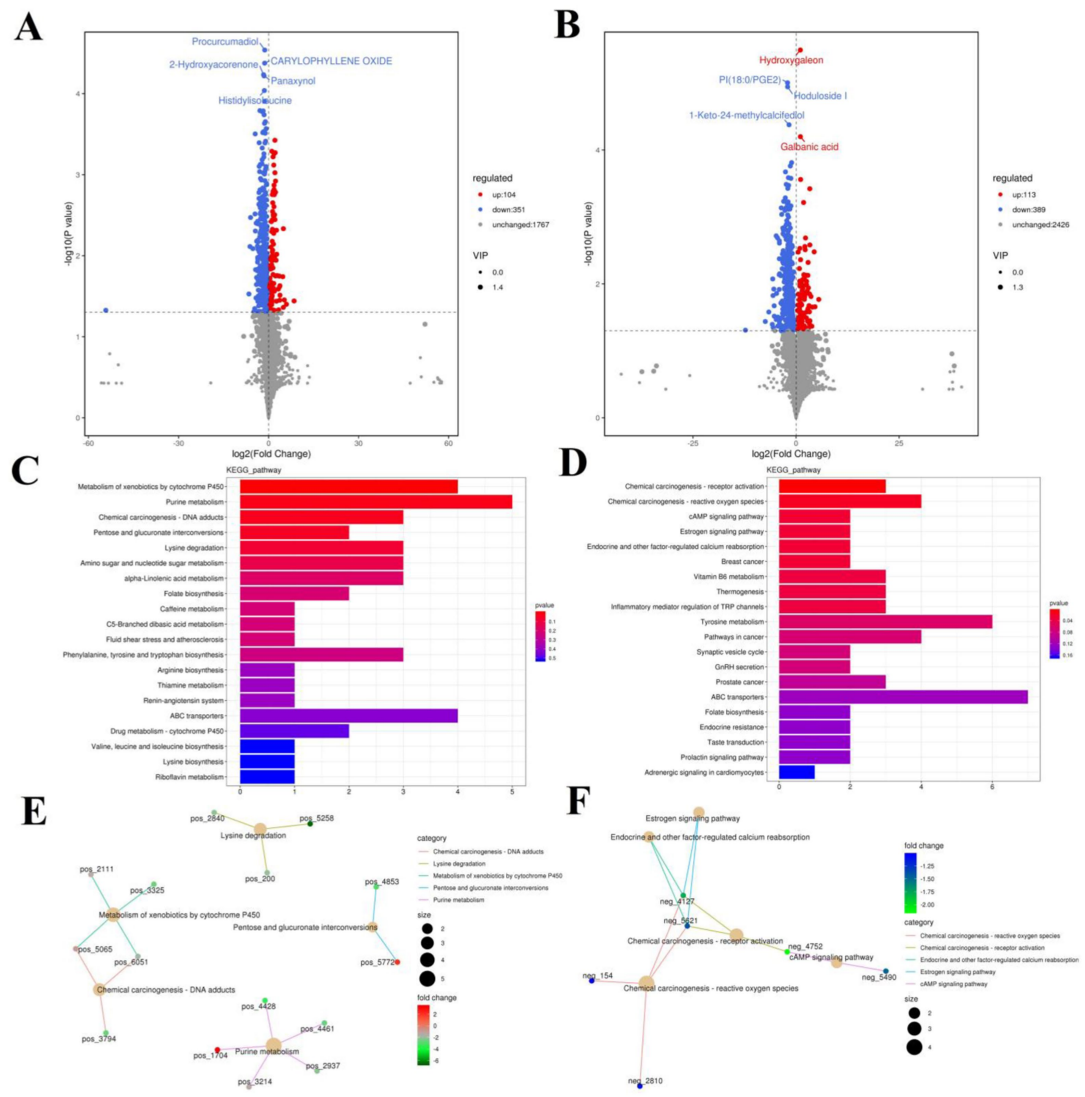
Effects of colitis on intestinal metabolism. **(A,B)** Volcano plot of differential metabolites between CON and DSS groups in positive-ion and negative-ion modes. The red dots represent the increased metabolites. The blue dots represent the decreased metabolites. **(C,D)** Intestinal metabolic pathways associated with colitis in positive-ion and negative-ion modes. **(E,F)** The representative network diagram of metabolites and related metabolic pathways between CON and DSS groups in positive-ion and negative-ion modes.

**Figure 9:**
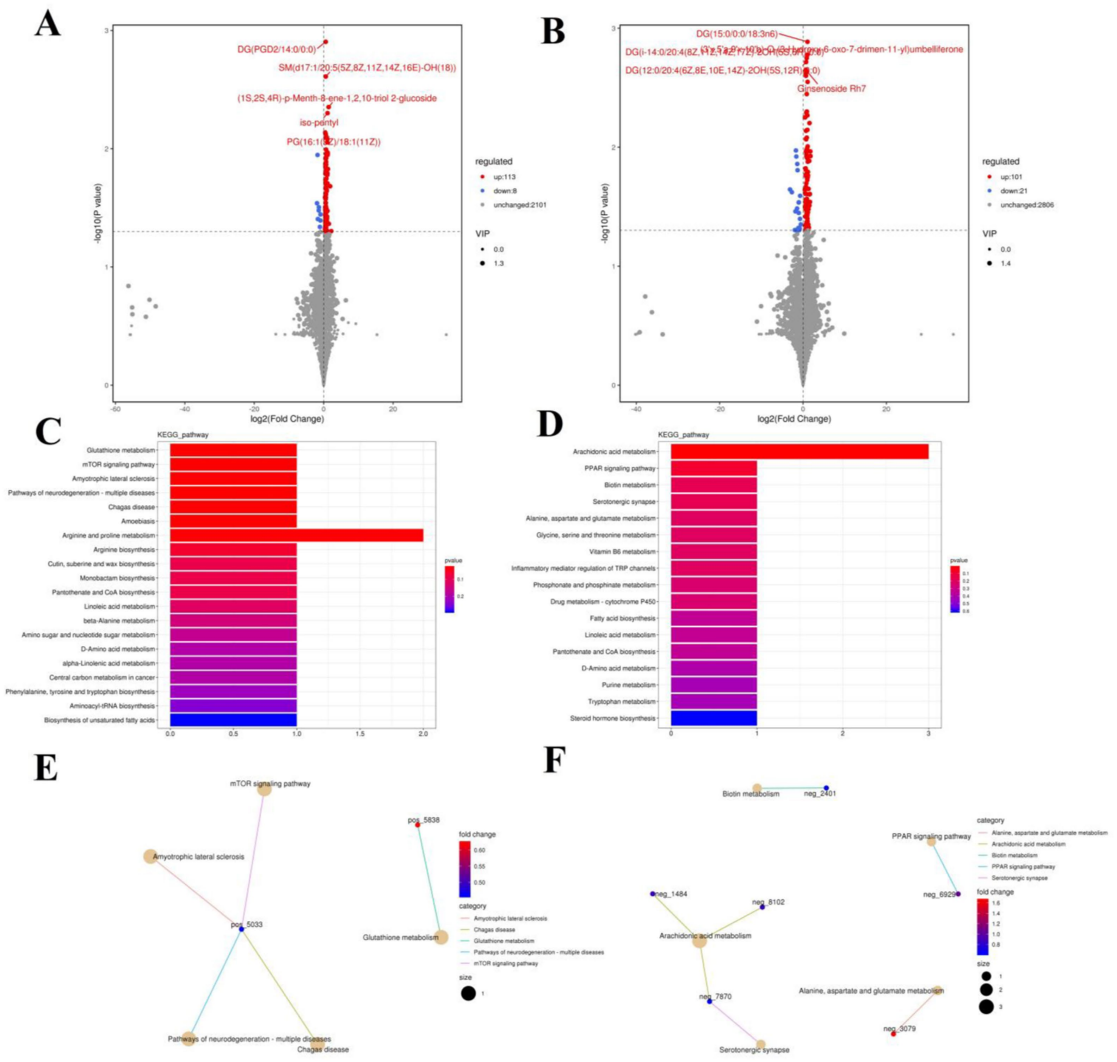
Effects of colitis on intestinal metabolism. **(A,B)** Volcano plot of differential metabolites between DSS and RSPQ groups in positive-ion and negative-ion modes. The red dots represent the increased metabolites. The blue dots represent the decreased metabolites. **(C,D)** Intestinal metabolic pathways associated with colitis in positive-ion and negative-ion modes. **(E,F)** The representative network diagram of metabolites and related metabolic pathways between DSS and RSPQ groups in positive-ion and negative-ion modes.

The PCA analysis indicated that colitis caused distinct changes in intestinal metabolism, while *Pediococcus acidilactici* administration ameliorated intestinal metabolism in mice with colitis (Figures 6, 7). To further reveal the positive regulation of *Pediococcus acidilactici* on intestinal metabolism, OPLS-DA score plots was applied for pattern discriminant analysis. Results indicated that there was a clear separation among CON, DSS, and RSPQ groups and no fitting occur. There are 957 (455 in positive mode, 502 in negative-ion mode) differential metabolites were detected between CON and DSS groups (Figures 8A,B). Among significantly different metabolites, 740 metabolites were down-regulated, whereas 217 metabolites were up-regulated in DSS group. Additionally, we observed that there were 1,018 metabolites exhibiting significant differences between the CON and RSPQ groups (Figures 9A,B). Among these, 208 (89 in positive mode, 119 in negative-ion mode) metabolites were significantly increased, while 810 (403 in positive mode, 407 in negative-ion mode) metabolites were significantly decreased in the RSPQ group compared to the CON group. For the comparison of the DSS and RSPQ groups, 243 metabolites (121 in positive mode, 122 in negative-ion mode) were totally identified, while the richness of 28 metabolites increased dramatically, whereas 215 metabolites showed the opposite trend.

The representative enriched pathways associated with colitis and *Pediococcus acidilactici* administration were present in Figures 8C,D and Figure 9C,D. Among enriched pathways between CON and DSS groups, 13 pathways with a significant difference were the metabolism of xenobiotics by cytochrome P450, purine metabolism, chemical carcinogenesis-DNA adducts, pentose and glucuronate interconversions, chemical carcinogenesis-receptor activation, chemical carcinogenesis-reactive oxygen species, cAMP signaling pathway, estrogen signaling pathway, endocrine and other factor-regulated calcium reabsorption, breast cancer, vitamin B6 metabolism, thermogenesis, inflammatory mediator regulation of TRP channels, which involved in 24 potential biomarkers including L-Noradrenaline, dimethylarsinous acid, oleoylethanolamide, 4-Pyridoxic acid, 2-Oxo-3-hydroxy-4-phosphobutanoate, 2-(Hydroxymethyl)-4-oxobutanoate, estradiol, 5-HETE, histamine, 15(S)-HETE, 5-Amino-4-imidazolecarboxyamide, dGMP, xanthosine, dIMP, Sudan I, digalacturonate, and D-Fructuronate, etc. (Table 1). Moreover, there were 8 pathways that were significantly different between the DSS and RSPQ, namely glutathione metabolism, mTOR signaling pathway, amyotrophic lateral sclerosis, pathways of neurodegeneration-multiple diseases, chagas disease, amoebiasis, arginine and proline metabolism, and arachidonic acid metabolism (Table 2). These pathways are linked to five potential biomarkers: spermine, L-Arginine, 15-Deoxy-Delta12,14-PGJ2, prostaglandin J2, and 15(S)-HETE. The metabolic diagrams related to colitis or *Pediococcus acidilactici* administration in the intestine were shown in Figures 8E,F and Figures 9E,F.

**Table 1:** Identification of potential biomarkers associated with differential metabolic pathways between the CON and DSS groups.

Pathway	Biomarkers	*p*	VIP	FC	Trend
Metabolism of xenobiotics by cytochrome P450	2-S-glutathionyl acetate	0.0020	1.76	0.38	Down
	1-Nitro-5-hydroxy-6-glutathionyl-5,6-dihydronaphthalene	0.03	1.41	0.15	Down
	1-(Methylnitrosoamino)-4-(3-pyridinyl)-1,4-butanediol	0.014	1.41	0.53	Down
	4-Oxo-1-(3-pyridyl)-1-butanone	0.027	1.41	0.27	Down
Purine metabolism	2-(Formamido)-N1-(5-phospho-D-ribosyl)acetamidine	0.046	1.39	9.98	Up
	5-Amino-4-imidazolecarboxyamide	0.00016	1.82	0.21	Down
	dGMP	0.049	1.36	0.39	Down
	xanthosine	0.018	1.46	0.095	Down
	dIMP	0.0015	1.77	0.15	Down
Chemical carcinogenesis - DNA adducts	Sudan I	0.048	1.31	0.151	Down
	1-(Methylnitrosoamino)-4-(3-pyridinyl)-1,4-butanediol	0.014	1.41	0.53	Down
	4-Oxo-1-(3-pyridyl)-1-butanone	0.027	1.41	0.27	Down
Pentose and glucuronate interconversions	Digalacturonate	0.015	1.58	0.12	Down
	D-Fructuronate	0.047	1.33	4.22	Up
Primary bile acid biosynthesis	Glycochenodeoxycholate	0.012	1.53	0.22	Down
	3beta,7alpha-Dihydroxy-5-cholestenoate	0.0079	1.69	0.17	Down
	7alpha-Hydroxycholesterol	0.043	1.36	0.61	Down
Neomycin, kanamycin and gentamicin biosynthesis	Nebramycin factor 4	0.010	1.70	0.33	Down
	2′-N-Acetylparomamine	0.026	1.47	1.96	Up
	4′-Oxolividamine	0.040	1.26	0.450	Down
Arachidonic acid metabolism	5-HETE	0.042	1.48	4.03	Up
	Prostaglandin C2	0.033	1.51	0.25	Down
	15(S)-HETE	0.024	1.43	1.60	Up
Valine, leucine and isoleucine degradation	L-Valine	0.0079	1.56	0.39	Down
Valine, leucine and isoleucine biosynthesis	L-Valine	0.0079	1.56	0.39	Down
Polyketide sugar unit biosynthesis	dTDP-4-oxo-5-C-methyl-L-rhamnose	0.044	1.40	0.077	Down
Glycerophospholipid metabolism	CDP-Ethanolamine	0.035	1.48	10.21	Up
Gap junction	L-Noradrenaline	0.018	1.56	0.22	Down
Aldosterone synthesis and secretion	Aldosterone	0.021	1.59	8.84	Up
Mineral absorption	L-Valine	0.0079	1.56	0.39	Down
Linoleic acid metabolism	12,13-DHOME	0.0095	1.68	2.44	Up
	13(S)-HODE	0.010	1.53	1.91	Up

Fold change (FC) was calculated by dividing the normalized means of metabolites in DSS by the CON.

**Table 2:** Identification of potential biomarkers associated with differential metabolic pathways between the RSPQ and DSS groups.

Pathway	Biomarkers	*p*	VIP	FC	Trend
Glutathione metabolism	Spermine	0.013	1.90	1.54	Up
mTOR signaling pathway	L-Arginine	0.031	1.72	1.37	Up
Amyotrophic lateral sclerosis	L-Arginine	0.031	1.72	1.37	Up
Pathways of neurodegeneration - multiple diseases	L-Arginine	0.031	1.72	1.37	Up
Chagas disease	L-Arginine	0.031	1.72	1.37	Up
Amoebiasis	L-Arginine	0.031	1.72	1.37	Up
Arginine and proline	Spermine	0.013	1.90	1.54	Up
metabolism	L-Arginine	0.031	1.72	1.37	Up
Arachidonic acid metabolism	15-Deoxy-Delta12,14-PGJ2	0.042	1.86	1.82	Up
	Prostaglandin J2	0.015	2.04	1.53	Up
	15(S)-HETE	0.010	2.21	1.81	Up

Fold change (FC) was calculated by dividing the normalized means of metabolites in DSS by the RSPQ.

## Discussion

The adverse effects of colitis on both human and animal health have attracted increasing attention (41). Although the specific pathogenesis of colitis has not yet been fully elucidated, it has been demonstrated to be closely associated with multiple factors such as environment, bacterial infection, oxidative stress, and genetics (42, 43). Recently, some studies involving gut microbiota have reported its important role in the development of colitis (44). Antibiotics remain the most commonly used treatment for colitis. However, antibiotics are no longer recommended for long-term use, due to the negative effects on hots health such as bacterial resistance, drug residues, and gut microbial imbalance. Probiotics have received widespread attention due to their important roles in gut microbiota, immunity, and metabolism (45, 46). Thus, *in vitro* supplementation of probiotics is also considered an effective strategy to alleviate colitis. In this study, we systematically explored the alleviating effect of *Pediococcus acidilactici* isolated from dogs on colitis using microbiome and metabolomics technologies.

To investigate the possible mechanism, we hypothesized that *Pediococcus acidilactici* administration could mediate the gut microbiota and their metabolites to further attenuate the colitis. Gut microbial composition and diversity were previously demonstrated to be associated with multiple intestinal diseases (47). In this study, colitis caused intestinal injury and decreased gut microbial alpha diversity, which is consistent with the findings of previous studies (47, 48). Early investigations suggested that intestinal injury inevitably could affecte intestinal environment and microbial survival, which in turn force existing microorganisms to adapt to new intestinal environment and alter the gut microbial composition and diversity, perturbing gut microbial homeostasis (49). The stable gut microbiota is an important biological barrier against the colonization and overgrowth of pathogens and conditioned pathogens (50). Conversely, gut microbial dysbiosis can affect intestinal mucosal barrier and immunologic function, thus increasing susceptibility to pathogens (50, 51). In this study, we observed that *Pediococcus acidilactici* administration could alleviate colon damage and reverse the reduction of microbial index caused by colitis.

We further dissected the relationship between colitis and gut microbiota and observed that colitis could cause changes in some specific bacteria that may play crucial roles in intestinal homeostasis and function. In this research, colitis cause a decline in the relative abundances of *Bacteroidetes*, *Prevotellaceae*, *Alloprevotella*, *Rikenellaceae*, *Alistipes*, and *Roseburia* and an increase in the proportion of *Proteobacteria*, *Turicibacter*, *Bilophila*, *Escherichia_Shigella*, and *Enterococcus*. Previous research indicated that the gut-residing *Bacteroidetes* is responsible for degrading carbohydrates and proteins (52). Meanwhile, it has been demonstrated to it can facilitate the development of the gastrointestinal immune system (53). As a pro-inflammatory bacterium, the levels of *Turicibacter* is significantly increased during enteritis (54). Earlier investigations showed that the increased *Bilophila* may contribute to the development of appendicitis and colitis (55). *Escherichia_Shigella* has been reported to be associated with diarrhea and intestinal infections (56). *Enterococcus* are common pathogens that can cause sepsis, pericarditis, and meningitis (57). Additionally, *Enterococcus* have been demonstrated to have both intrinsic and acquired drug resistance, which severely affects the treatment of their infections (58). *Prevotellaceae* has been shown to possess the ability to degrade polysaccharide and high-carbohydrate substrates (59). Thus, the decreased abundance of *Prevotellaceae* in the intestine may negatively impact host nutrient uptake. *Alloprevotella* has been demonstrated to be closely related to the decreased lifetime cardiovascular disease risk due to its ability to produce acetate and succinate (4, 5). As a common beneficial intestinal bacteria, *Rikenellaceae* could degrade plant-derived polysaccharides and alleviate the colitis by mediating T-regulatory cell differentiation (60). *Alistipes* and *Roseburia* are potential producers of SCFAs. Previous investigations involving SCFAs have indicated their vital roles in immune system, cell proliferation, energy intake and intestinal metabolism (61, 62). Additionally, SCFAs also participate in the positive regulation of gut microbiota and intestinal barrier, which are essential for host health (63). However, *Pediococcus acidilactici* administration can improve the gut microbial composition of patients with colitis. Notably, *Pediococcus acidilactici* administration can increase the relative abundances of *Akkermansia*. *Akkermansia* has long been recognized as a beneficial intestinal bacterium, and its abundance gradually decreases with the development of enteritis (64). Numerous studies indicated that *Akkermansia* can alleviate intestinal inflammation and prevent intestinal cancer by regulating the immune response in the spleen, intestines, and mesenteric lymph nodes (65, 66). Moreover, *Akkermansia* has been demonstrated to be negatively related to obesity and diabetes (67, 68). These results showed the vital roles of *Pediococcus acidilactici* administration in regulating gut microbiota, which contributed to maintaining intestinal colonization resistance and homeostasis.

Previous studies indicated that the gut microbiota can systematically regulate host health by producing metabolites. Therefore, we also explored the changes in intestinal metabolism. In this study, we observed that eight metabolic pathways (glutathione metabolism, arginine and proline metabolism, arachidonic acid metabolism, etc.) and 5 (spermine, L-Arginine, 15-Deoxy-Delta12,14-PGJ2, prostaglandin J2, and 15(S)-HETE) metabolites were involved in the alleviating effect of *Pediococcus acidilactici* administration. These significantly changed metabolites and metabolic pathways may play a key role in *Pediococcus acidilactici* alleviating colitis. Previous studies have demonstrated that arachidonic acid can alleviate oxidative stress by increasing the levels of superoxide dismutase (SOD) and catalase (CAT) (69). Furthermore, arachidonic acid has been shown to inhibit oxidative stress by reducing mitochondrial membrane potential and blocking reverse electron transport (RET) through uncoupling, which in turn inhibits RET-dependent reactive oxygen species (ROS) generation (70). As an important intracellular metabolic regulator and antioxidant, glutathione can participate in the tricarboxylic acid cycle and sugar metabolism in the body (71). It can also activate multiple enzymes, thereby facilitating the metabolism of carbohydrates, fats and proteins. Additionally, glutathione play a crucial role in maintaining the function of the immune system and the stability of the red blood cell membrane structure (72). Recent research indicates that glutathione can rectify the imbalance of acetylcholine and cholinesterase, exert an anti-allergic effect, and enhance the skin’s antioxidant capacity (73). Previous research has indicated that oxidative stress is a significant factor contributing to the development of colitis. Thus, *Pediococcus acidilactici* may alleviate the development of colitis by mediating arachidonic acid metabolism and glutathione metabolism.

## Conclusion

Taken together, this study investigated the alleviating effect of *Pediococcus acidilactici* on colitis. Results indicated that *Pediococcus acidilactici* could alleviate colonic injury and reverse the decline in gut microbial diversity and abundance associated with colitis. Additionally, we also observed a positive effect of *Pediococcus acidilactici* on intestinal metabolism in patients with colitis. Arachidonic acid metabolism and glutathione metabolism may be potential pathways for *Pediococcus acidilactici* to exert its effects. This study is an important exploration of whether *Pediococcus acidilactici* isolated from dogs can alleviate colitis. Meanwhile, it also contributes to expanding the probiotic application and provide a theoretical basis for alleviating colitis from the microbial and metabolic perspective. However, it is important to acknowledge the limitations of this study, particularly the small sample size.

## Data Availability

The original sequence data was submitted to the Sequence Read Archive (SRA) (NCBI, USA) with the accession no. PRJNA1179910.
