# Supplementary figures and images for: Integrated microbiome and metabolomics analysis reveals the alleviating effect of Pediococcus acidilactici on colitis
Source: Front Vet Sci. 2025 Feb 26;12:1520678. doi: 10.3389/fvets.2025.1520678 (PMC11897304; doi:10.3389/fvets.2025.1520678)

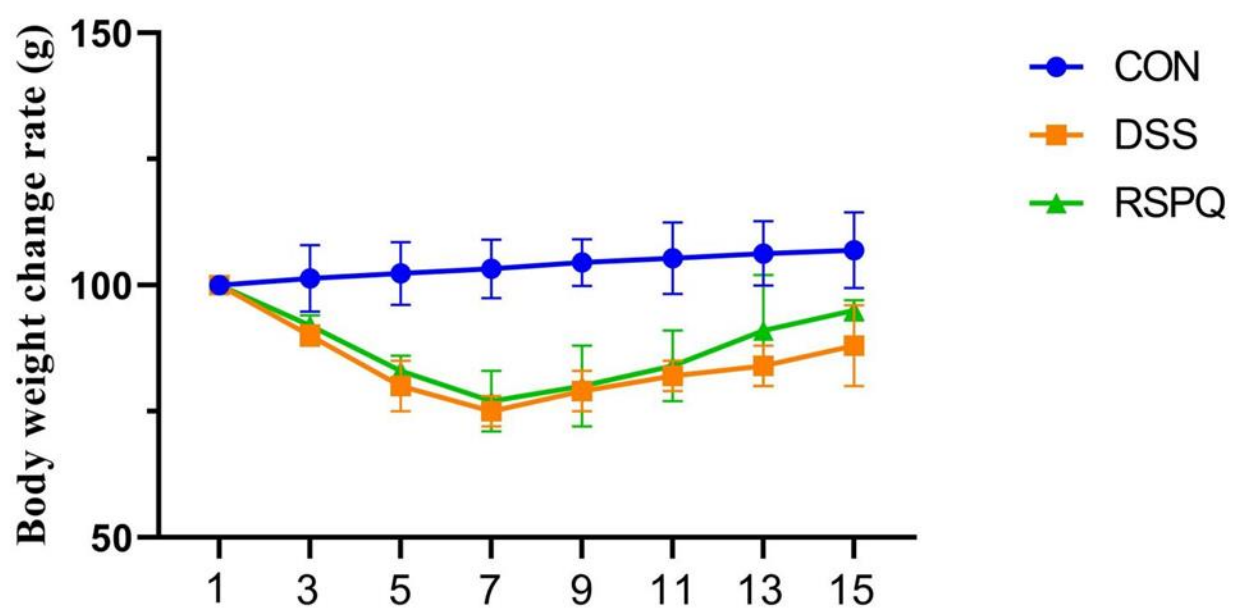

**Supplementary Fig. S1.** The rate of body weight change throughout the experiment.

Supplement: Supplementary file 1 [file Image_1.pdf]
